# Single-institution case series of pituitary biopsy for suspected germinoma in the pediatric population: diagnostic utility, operative risks, and biopsy approaches

**DOI:** 10.1038/s41598-020-71988-7

**Published:** 2020-09-17

**Authors:** Emily L. Day, Edward R. Smith, Katie P. Fehnel

**Affiliations:** Department of Neurosurgery, Boston Children’s Hospital, 300 Longwood Avenue, Boston, MA 02115 USA

**Keywords:** Germ cell tumours, Paediatric cancer

## Abstract

Little has been reported on the safety and efficacy of pituitary biopsy in the pediatric population for suspected germinoma. An updated review is needed. Patients who underwent biopsy (endoscopic endonasal vs. open craniotomy) for isolated pituitary stalk thickening were identified. Age, pre- and post-operative endocrine status, surgical approach, length of surgery, estimated blood loss, surgical morbidity, length of ICU stay, total length of stay, and pathology reports were reviewed. Nine patients met inclusion criteria. Germinoma diagnosis was rendered in 7 of 9 patients; 1 patient required two biopsy attempts. Two-patients had histology consistent with inflammation and a subsequently self-limited disease course. Average operative time, blood loss, ICU stay and overall length of stay was just over 2 h, 28 mL, 1.6 days and 3.7 days respectively. There were no intraoperative complications and all patients were discharged home. One patient developed new diabetes insipidus post-operatively. Patients who underwent endoscopic biopsy had decreased operative times and shorter hospitalizations. Biopsy for isolated pituitary stalk thickening for suspected germinoma is generally safe with high diagnostic utility. Importantly, 22% of presumed germinomas on imaging yielded alternative diagnoses on biopsy, adding support for pathology-proven data to guide treatment in relevant cases.

## Introduction

Isolated pituitary stalk thickening and/or progressive thickening in setting of pituitary dysfunction in the pediatric population is often most worrisome for germ cell tumor, specifically germinoma. Central nervous system (CNS) germ cell tumors (GCT) are rare and account for < 5% of all CNS tumors and < 10% of pediatric brain tumors^1–6^. Of these, germinomas comprise approximately two thirds of all CNS germ cell tumors^1,7,8^ and are commonly located either in the pituitary or pineal gland^1,2,6,8,9^.

In patients for whom a diagnosis of germ cell tumor is suspected, the standard approach has been to obtain intracranial imaging, to sample serum and cerebral spinal fluid (CSF) for tumor markers (AFP and bHCG), and to have baseline endocrine and ophthalmologic evaluations. If diagnosis cannot be obtained via serum and CSF markers, a tissue diagnosis is needed to guide treatment^1,8,10,11^. The diagnostic algorithm for GCTs affecting the pineal recess has been more standardized than for the pituitary-specific lesions due to the fact these patients often present with hydrocephalus from mass effect on the posterior third ventricle with aqueductal obstruction. Therefore, for this subset of patients, in the setting of negative tumor markers, surgical treatment includes a combination of CSF diversion by endoscopic third ventriculostomy and endoscopic biopsy of the mass in the posterior third ventricle^12^.

In contrast, there is no comparable standardized approach to germ cell tumors involving the pituitary gland and pituitary stalk. Our institution recommends biopsy for all patients with diabetes insipidus and pituitary stalk thickening on MRI (defined as > 2.6 mm) in the setting of either anterior pituitary dysfunction or with evidence of progressive stalk thickening on serial pituitary imaging^13,14^.

There is no existing literature to estimate the relative risk or efficacy of biopsy in this population.

Often approached by craniotomy, these are considered technically challenging biopsies with high risk of morbidity and potentially non-diagnostic tissue. This risk, coupled with the relative accessibility of the gland and stalk via transsphenoidal approach, led us to consider an endoscopic transsphenoidal approach as an alternative to craniotomy for some of these cases. This study seeks to determine the overall efficacy and safety of pituitary stalk biopsy in our institution in the setting of suspected germinoma as well as to compare the different operative approaches with respect to diagnostic yield and operative outcomes.

Here we retrospectively review all patients for whom a pituitary stalk biopsy was indicated based on the aforementioned criteria and report the primary outcomes of diagnostic yield (% confirmed pathology) and operative morbidity as well as secondary outcome measures pertaining to surgical morbidity with respect to open versus endoscopic biopsy, with the paired goals of (1) demonstrating the efficacy and risk of the transsphenoidal approach versus craniotomy and (2) generating specific inclusion criteria that may help surgeons to appropriately select patients for the transsphenoidal approach when faced with this clinical scenario.

## Methods

### Patient selection (Fig. 1)

**Figure 1:**
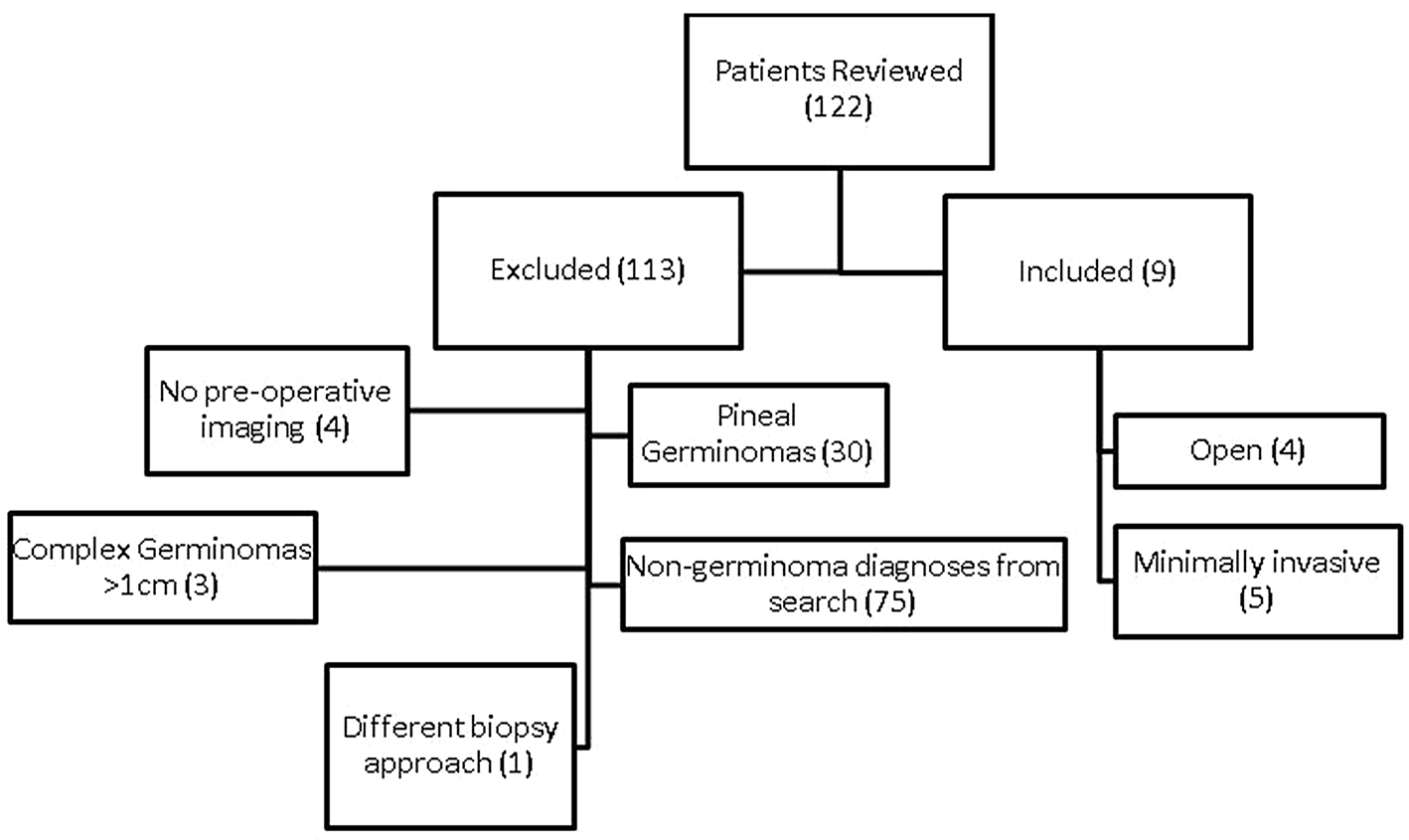
Inclusion and exclusion criteria. Shown is a flowchart demonstrating flow of patient selection based on defined inclusion and exclusion criteria.

The institutional review board of Boston Children’s Hospital approved this study (IRB-P00027869); this IRB allows for informed consent to be waived on retrospective reviews. All methods were carried out in accordance with relevant guidelines and regulations. An electronic search to identify isolated pituitary/infundibular lesions at presentation was performed in our institutional surgical database using search terms “pituitary,” “infundibulum,” “germinoma,” “germ cell tumor” or “diabetes insipidus” to identify subjects < 21 years of age at time of diagnosis. All patients were evaluated and operated on by one of 4 surgeons in the Department of Neurosurgery at Boston Children’s Hospital between August 1998 and May 2019. Specifically, the open biopsies were performed from August 1998 to February 2017; the endoscopic biopsies were performed from July 2013 to May 2019. 122 patients were identified over a 20 year period. Patients were excluded based on other diagnosis (e.g. craniopharyngioma, adenoma, glioma, other), location (pineal), lesion size > 1 cm with suprasellar extension, incomplete clinical data or absent preoperative imaging. Nine patients met criteria for inclusion and were additionally subdivided by biopsy approach—open craniotomy versus endoscopic (transphenoidal/endoscopic endonasal).

### Data collection

We retrospectively reviewed the electronic medical records for all patients included. Variables assessed at time of presentation included demographics, age, endocrine status (hypothyroidism, growth hormone deficiency, gonadotropic deficiency, adrenal insufficiency, precocious puberty, delayed puberty, hyperprolactinemia, and diabetes insipidus (DI)), presenting symptoms, ophthalmologic baseline, imaging characteristics, serum and CSF tumor markers (AFP, bHCG), and any prior tumor treatment. Perioperative variables assessed included surgical procedure (open craniotomy vs. endoscopic biopsy), diagnostic yield, length of surgery, estimated blood loss (EBL), post-operative endocrine status, length of ICU stay, total length of stay, surgical morbidity, and operative complications including CSF leak, vascular injury, stroke, neurologic deficit, worsened visual acuity, pituitary or hypothalamic dysfunction.

### Imaging review

All available Magnetic Resonance Imaging (MRI) and Computed Tomography (CT) was reviewed for all patients. In addition to lesion size and location, infundibular size/stalk thickness, presence or absence of pituitary bright spot, intracarotid distance, extent of sinus pneumatization, sella angle, and ventricular size were documented based on pre-operative imaging (Table 1).

**Table 1:** Summary of presenting endocrine dysfunction.

	Open (%) (n = 4)	Endoscopic (%) (n = 5)	Total (%) (n = 9)
**Endocrine**	100	100	100
Hypothyroidism	50	40	44
Growth hormone deficiency	25	40	33
Gonadotropic deficiency	25	20	22
Adrenal insufficiency	25	20	22
Precocious puberty	25	0	10
Delayed puberty	25	0	10
Hyperprolactinemia	25	0	10
Diabetes insipidus (DI)	50	100	80
**Headaches**	50	66	78

### Statistical analysis

Patients were designated as open versus endoscopic. An independent samples t-test was performed using SPSS (version 24.0, IBM Corporation, Armonk, NY) to detect significance (*P* < 0.05) among variables such as length of surgery, length of stay, length of ICU stay, and estimated blood loss (EBL).

## Results

### Demographics

A total of nine patients (3F:6M) were identified as presenting initially with isolated pituitary/infundibular abnormalities ranging in age from 3 to 15 years. No patient had previously undergone operative intervention or biopsy and only one patient had been empirically treated with chemotherapy prior to undergoing biopsy for medically refractory tumor progression.

### *Presentation* (Table 1)

Out of 9 patients included, 55% presented with headaches and all 10 presented with endocrine dysfunction. The majority—77% had had diabetes insipidus (DI) at time of presentation, 44% had hypothyroidism, 33% had growth hormone deficiency, 22% had adrenal insufficiency, 22% had gonadotropic deficiency, 22% had either delayed or precocious puberty, and one patient had elevated prolactin. One patient had non-specific ophthalmologic symptoms. No patient had elevated AFP or bHCG (serum or CSF).

### *Imaging characteristics* (Table 2)

**Table 2:** Summary of presenting demographics, operative data, and diagnostic data.

Case	Sex	Age (yrs.)	Tumor markers	Stalk (mm)	Intracarotid distance (mm)	Pneumatized sinuses	Ventriculomegaly	Biopsy	Operative time (h)	Pathologic diagnosis	LOS (days)	ICU stay (days)
A	M	7.3	Normal	6	14	Y	N	Open	N/A	Lymphocytic infiltrate	4	1
B	M	15.4	Normal	5	15	Y	N	End	3:26*	Germinoma	4	1
C	M	13.7	Normal	8	20	Y	N	End	2:12	Germinoma	2	1
D	F	9.5	Normal	6	13.5	Incomplete	N	Open	4:12	Germinoma	7	2
E	M	13.2	Normal	4.5	20	Y	N	End	1:39	Germinoma	2	1
F	F	6.9	Normal	3.5	14	Y	N	End	2:39*	Germinoma	2	1
G	M	8.0	Normal	5	14	Y	N	Open	N/A	Neuroglial tissue with rare lymphoid cells	4	2
H	F	13.5	Normal	8	16	Y	N	End	2:16*	Anterior pituitary with scant lymphoid infiltrate	3	1
I	M	4.0	Normal	4	18	Incomplete	N	Open	5:08	Germinoma	5	4

End, Endoscopic surgery; N/A, data not available.

*Joint with otolaryngology.

All patients underwent magnetic resonance imaging (MRI) which demonstrated uniform absence of the pituitary bright spot in all 9 patients and pituitary stalk thickening of > 2.6 mm all of the patients. No patient had hydrocephalus. One patient had ventriculomegaly. Seven out of the nine patients had pneumatized sphenoid sinuses. Intracarotid distance was > 13.5 mm for all patients who underwent transphenoidal biopsy.

### Operative approach

#### Open craniotomy versus endoscopic endonasal

Open craniotomy was performed through a standard pterional craniotomy and subfrontal approach for stalk biopsy. Endoscopic endonasal biopsy was performed via standard transphenoidal approach to the sella for sampling of the pituitary gland^15^. Briefly, using Mayfield pin fixation, the patient was positioned supine with slight rightward head turn, care taken to avoid venous compression and therefore maintained in neutral to slight extension. Using frameless stereotaxy, bimanual endoscopic endnonasal approach to the sella via the sphenoid sinus was utilized. The pituitary gland was split vertically to facilitate sampling of the posterior gland and frozen specimen was taken to confirm lesional tissue. Additional biopsies were taken as appropriate.

#### Patient selection (Tables 2 and 4)

Operative approach was determined by the surgeon. On average, the patients for whom an endoscopic endonasal biopsy was attempted were older with an average age at surgery of 12.6 ± 3.0 years versus 7.2 ± 2.0 years for open craniotomy.

All patients for whom endoscopic transphenoidal approach was performed shared the following imaging findings:

1. Pneumatized sphenoid sinus
2. Intracarotid distance of > 1 cm
3. Stalk thickening of 3.5 mm or greater
4. Involvement of the abnormal thickening down to (and contiguous with) the pituitary gland on sagittal MRI studies.

The imaging features and patient characteristics were otherwise comparable between the two populations.

#### Overall surgical success (Tables 2 and 3)

**Table 3:** Operative and perioperative data.

	All cases (n = 9)	Open (n = 4)		Endoscopic (n = 5)	*P* value (endoscopic vs. open)
Age at surgery (yrs.)	10.21 [3.77]	7.18	[2.00]	12.63 [3.04]	***0.03***
**Sex**					N/A
Female	3 (33%)	1	(25%)	2 (40%)	
Male	6 (66%)	3	(75%)	3 (60%)	
Diagnostic success	6 (66%)	2	(50%)	4 (80%)	0.125
Operative time (min.)	184.57 [69.01]	280	[28]	146 [35.2]	***0.01***
Estimated blood loss (mL)	28 [22.99]	40	[23.45]	18 [17.20]	0.196
Complications	1 (11%)	1*	(25%)	0	*0.24*
Length of ICU stay (days)	1.6 [1.0]	2.3	[1.09]	1.0 [0]	***0.05***
Length of hospital stay (days)	3.7 [1.6]	5.0	[1.20]	2.6 [.8]	***0.01***

Categorical data are represented as no. (%).

Continuous data are represented as mean [SD].

An independent samples t-test was performed using SPSS (version 24.0, IBM Corporation, Armonk, NY) to detect significance (*P* < .05).

*new Diabetes Insipidus.

Of the 9 patients included, a pathology-confirmed diagnosis of germinoma was rendered in 77% of the biopsies. One patient with initially non-diagnostic open biopsy underwent repeat craniotomy with pathology-confirmation of germinoma. Two-patients had histology consistent with inflammation and a subsequently self-limited disease course. Average operative time was just over 2 h, average blood loss was 28 mL, average ICU stay was 1.6 days and average length of hospital stay was 3.7 days. Comparing the endoscopic versus open patients respectively, we found that there was a statistically significant decrease in average operative time (*p* = *0.01*; 146.4 ± 35.4 min vs. 280 ± 28 min), length of ICU stay (*p* = *0.05*; 1.0 ± 0.0 day vs. 2.3 ± 1.1 days) and overall length of stay (*p* = *0.01*; 2.6 ± 0.8 days vs. 5.0 ± 1.2 days) in the endoscopic biopsies. Additionally, there was a greater percentage of pathology-confirmed diagnosis in the endoscopic cohort as compared to the open craniotomies (80% endoscopic vs. 50% open). Of note, halfway through the study period the transphenoidal biopsies were performed with otolaryngology; in the first two cases with combined ORL and neurosurgery longer operative times reflect adjustment to the new work-flow.

### Surgery and follow-up

There were no intraoperative complications, no CSF leaks, and no patient deaths during the follow-up period. There were no new neurologic deficits or operative morbidities associated with the endoscopic biopsies, however there was one patient who developed diabetes insipidus following open craniotomy for stalk biopsy. Following surgery, all patients were discharged home, rather than to an outside facility. Of the 3 patients for whom biopsy was non-diagnostic, two patients elected not to pursue additional diagnostic testing and one patient underwent a repeat craniotomy as noted above.

## Discussion

Isolated pituitary stalk thickening and/or progressive thickening in setting of pituitary dysfunction in the pediatric population can be seen in setting of germ cell tumor, specifically germinoma. In patients for whom a diagnosis of germ cell tumor is suspected, the standard approach has been to obtain intracranial imaging, to sample serum and cerebral spinal fluid (CSF) for gold standard tumor markers (AFP and bHCG) and less commonly placental alkaline phosphatase (PLAP)^10–13^, and to have baseline endocrine and ophthalmologic evaluations. If diagnosis cannot be obtained via serum and CSF markers, a tissue diagnosis is needed to guide treatment^1,8,10,11,16^. There has been literature to suggest indications for biopsy in the setting of diabetes insipidus and pituitary stalk thickening on MRI in the setting of either anterior pituitary dysfunction or with evidence of progressive stalk thickening on serial pituitary imaging^13,16^, however there is no existing literature with respect to the risks and efficacy of biopsy in this setting.

Given the lack of literature to guide surgical management in this rare diagnosis, we reviewed our institutional experience. To our knowledge this is the largest pediatric surgical series of isolated pituitary germinomas in the literature. Here we identify nine pediatric patients with a suspected diagnosis of germinoma between 1998 and 2019, of which seven ultimately had pathologic confirmation.

Overall, we report a 77% rate of pathology-confirmed diagnosis from biopsy of any sort (7 out of 9 patients). Of the three patients for whom no diagnosis was primarily obtained (two open, one endoscopic), two underwent no additional biopsy and no further treatment and one patient underwent a second craniotomy with germinoma diagnosis confirmed at reoperation. Overall, the average length of stay was 3.7 days total and 1.6 days in the ICU, there were no operative complications aside from progression to DI in one patient, and all patients were discharged to home.

We sought to further study the difference between operative approaches with respect to both diagnostic utility and perioperative care to determine if there was an advantage to endoscopic sampling in this disease population. Though it failed to achieve statistical significance given the small sample size, this comparison demonstrated that compared to open craniotomy, patients who underwent endoscopic biopsy had an increased likelihood of obtaining pathology confirmation, and more strikingly a shorter length of surgery, and reduced length of both ICU and overall stay. This could be due to the nature of an endoscopic approach being naturally less invasive, but nonetheless it seems to provide more benefit to the patient in various ways^17^. It is worth noting that the length of stay difference is likely influenced by the patient for whom new post-operative DI necessitated 7 days in the hospital, however excluding that patient, the average LOS for craniotomy was 4.3 days versus an average of 2.6 days for endoscopic biopsy. To better understand the patients for whom endoscopic biopsy can safely be done, we analyzed the imaging characteristics of all patients. Successful transsphenoidal biopsy was performed in patients with pneumatized sinuses, an intracarotid distance of > 1 cm, and stalk thickening of at least 3.5 mm. Consequently, we identified criteria for consideration of transsphenoidal biopsy in cases of infundibular thickening (Table 4).

**Table 4:** Radiographic criteria for consideration of transphenoidal biopsy.

Pneumatized sphenoid sinus
Intracarotid distance of > 1 cm
Stalk thickening of 3.5 mm or greater
Abnormal thickening down to (and contiguous with) the pituitary gland on sagittal MRI studies

In other parts of the world, patients are often treated empirically for a radiographic diagnosis of germinoma, which has made extrapolation from large existing series rather limited as a means of answering this clinical question. For example, a recent large series published out of China reported 170 patients age < 40 years who were treated presumptively as germinomas. Of these, 95 patients were identified with a sellar/suprasellar tumor, however no patient in this series had pathologic diagnoses and in this combined pediatric/adult cohort all were treated empirically^18^.

While the primary goal of our study was to assess the technical aspects related to the safety and utility of the transsphenoidal approach, we were surprised to discover that 22% (2/9) patients did not harbor germinoma, despite a compelling radiographic presentation, a much higher number than expected from the literature. Of the two patients in our series who did not undergo additional biopsy and who were managed with observation alone, lesions remained stable at the last documented follow-up up to 2 years post biopsy attempt. Given the self-limiting disease course in these 2 patients, these simply may not be germinomas rather than biopsy failures. These data suggest that further research into the accuracy of imaging-only diagnosis without pathologic confirmation (as reported by other institutions) may lead to unnecessary treatment. Acknowledging the small sample size of our study, we would nonetheless propose that additional study into this potentially important finding could expand the role of surgical biopsy, particularly if endoscopic approaches are lower risk than craniotomy and add the benefit of avoiding unnecessary germinoma therapy in a substantial number of patients.

This is a limited sample size due to the rarity of this diagnosis with a focus on a subset of patients within this rare category. The retrospective nature of this study may result in patients who were excluded on the basis of misclassification given that germinoma does not have a specific ICD-9 or ICD-10 code and our surgical database search criterion may potentially miss patients who would otherwise meet inclusion criteria. The two patients for whom no definitive diagnosis was obtained more than likely represent non-germinoma diagnoses NOS though there is no means of confirming that in this study. With respect to the preferred operative approach for biopsy, the sample size here is too limited to adequately power that question and it should also be noted that there is surgeon bias. Our institutional bias has been towards endoscopic approaches over the past 15 years with particular expertise in that approach; it is possible that some of the patients who underwent open craniotomy would have had comparable outcomes via the endoscopic approach and vice versa. Future studies should be conducted with larger patient cohorts to further identify statistically significant variables.

## Conclusions

We conclude from this limited cohort that in patients for whom biopsy is indicated it may be attempted with at least a 77% chance of obtaining pathology-confirmed diagnosis regardless of approach, with limited operative morbidity and with an improved risk benefit profile that slightly favors endoscopic approaches in the appropriate patients. Given that 20% of patients in this study had a self-limiting course without evidence of germinoma at time of biopsy, the limited morbidity of biopsy may outweigh the risk of empiric treatment in patients with suspected but not biopsy proven germinoma.
